# Nicotianin-I: A Tobacco Floral Nectar Peptide with Anticandidal Activity

**DOI:** 10.1021/acsomega.4c10806

**Published:** 2025-05-14

**Authors:** João M. M. Neto, Tawanny K. B. Aguiar, Mariana F. Oliveira, Queilane L. S. G. Chaves, Dário R. A. L. Mourão, Viviane O. Silva, Maria T. V. Nascimento, Rômulo F. Carneiro, Rafael X. Martins, Davi F. Farias, Brandon F. Sousa, Jeanlex S. Sousa, Márcio V. Ramos, Cleverson D. T. Freitas

**Affiliations:** † Department of Biochemistry and Molecular Biology, Federal University of Ceará, Pici Campus, Fortaleza-Ceará CEP 60440-554, Brazil; ‡ Department of Biology, Federal University of Ceará, Pici Campus, Fortaleza-Ceará CEP 60440-554, Brazil; § Department of Fishing Engineering, Federal University of Ceará, Pici Campus, Fortaleza-Ceará CEP 60440-554, Brazil; ∥ Laboratory for Risk Assessment of Novel Technologies (LabRisk), Department of Molecular Biology, Federal University of Paraiba, Campus I, João Pessoa CEP 58051-900, Brazil; ⊥ Biological Physics Laboratory, Physics Department, Federal University of Ceará, Pici Campus, Fortaleza-Ceará CEP 60440-554, Brazil

## Abstract

Multidrug-resistant microorganisms are major threats to society, leading to the necessity of alternative molecules to fight them back. Antimicrobial peptides (AMPs), especially those derived from plants, have become relevant for multiple reasons. Therefore, this study evaluated six peptides identified in the floral nectar of ornamental tobacco for their effectiveness against four clinically relevant yeast species (Candida albicans, Candida krusei, Candida parapsilosis, and Candida tropicalis). Pep6 (KHYSCTRHGYCLACYKRWF) was the only one that showed activity against all yeast species, with IC_50_ values ranging from 24 ± 1.9 μM to 80 ± 4.6 μM. Pep6 (named nicotianin-I) was able to alter permeability or generate pores in the cell membranes of all microorganisms, in addition to inducing an overproduction of reactive oxygen species (ROS). Nicotianin-I adopted a structure similar to that of the polyproline II (PPII) helix in buffer and sodium dodecyl sulfate (SDS) and an α-helical pattern when subjected to a membrane mimetic environment (50% TFE). It demonstrated negligible hemolytic activity and no cytotoxic effects on murine fibroblast cells. Only at the highest concentration assessed (100 μg/mL or 42 μM), nicotianin-I caused a 40% mortality rate in zebrafish embryos and delayed their hatching. This study demonstrates the potential of a novel peptide with anticandidal activity and opens the door to the rational design of new analogous peptides with improved properties.

## Graphical abstract

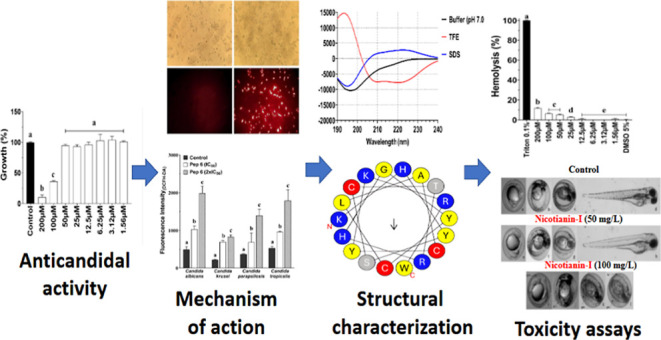

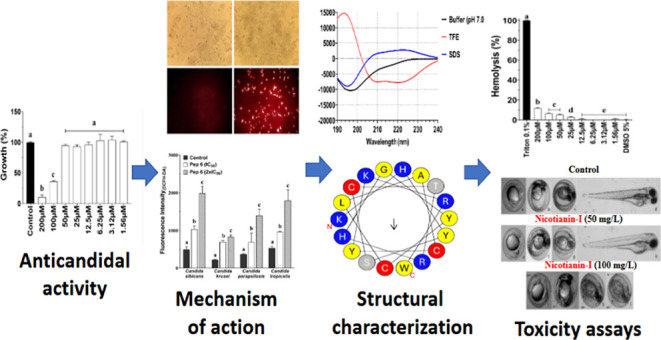

## Introduction

Pathogenic microorganisms, including bacteria, filamentous fungi, and yeasts, are responsible for a significant number of hospitalizations and fatalities worldwide. In 2019, infectious syndromes accounted for more than 13 million deaths.^1^ This scenario has intensified largely due to the emergence of new pathogenic strains that exhibit treatment resistance.^2^ Yeasts, particularly those of the Candida genus, have garnered significant attention as pathogens implicated in infectious diseases.^3^

Candida species-mediated infection can be classified into two subtypes: (1) candidiasis, which is characterized primarily by yeast proliferation on mucosal tissues, such as the oral mucosa, gastrointestinal tract, and vaginal mucosa; and (2) candidemia, which is a systemic type of candidiasis that affects multiple organs and is associated with a high mortality rate.^3,4^ Furthermore, the emergence of clinical isolates resistant to available antifungal drugs can exacerbate these threats.^5^

Consequently, some surveys have been developed to discover novel therapeutic options that can surpass and outperform the existing ineffectiveness of antifungal antibiotics.^6^ Antimicrobial peptides (AMPs) have distinguished themselves in this aspect since they are recognized for their amphiphilic α-helices and overall positive charge. These characteristics have been associated with their primary mechanisms of action, including damage to the cell membrane and inducing oxidative stress by overproducing reactive oxygen species (ROS).^7^

Plant antimicrobial peptides possess biochemical properties that make them highly favorable candidates for the treatment of human infections.^8^ These AMPs are crucial elements of the innate immune system and are present in various plant tissues and organs, such as leaves,^9^ seeds,^10^ and others. Floral nectar has recently been identified as an abundant biological source of AMPs. Parra and colleagues^11^ conducted the sequencing and characterization of almost 800 peptides derived from the floral nectar of ornamental tobacco. Afterward, they selected six peptides and proved their ability to kill some phytopathogenic fungi and bacteria. Due to their high biotechnological potential, the current study focuses on the use of these six tobacco floral nectar peptides as anticandidal agents. Therefore, we describe the in vitro antifungal potential of these six peptides against Candida albicans, Candida krusei, Candida parapsilosis, and Candida tropicalis, with a focus on their mechanisms of action. Moreover, we characterized by circular dichroism (CD) and performed in vitro and in vivo toxicity tests with the most promising peptide.

## Results and Discussion

### Anticandidal Activity

The present study aimed to assess the anticandidal properties of six peptides derived from ornamental tobacco floral nectar (a cross of Nicotiana sanderae and Nicotiana langsdorffii). The study specifically targeted C. albicans, C. krusei, C. parapsilosis, and C. tropicalis, assessing both the inhibitory capabilities and mechanisms of action. With respect to the in vitro antifungal activity, peptides Pep1, Pep2, Pep3, Pep4, and Pep5 exhibited negligible or no significant inhibition against all strains tested (Figure 1). On the other hand, peptide Pep6 not only suppressed the growth of all tested organisms but also showed the lowest concentrations of inhibition among other peptides that were anticandidal (Figure 1). The respective IC_50_ and MIC values for peptide Pep6 against the Candida species were as follows: 80 ± 4.6 μM (194 μg/mL) and 120 ± 9.3 μM (292 μg/mL) for C. albicans, 57 ± 3.7 μM (140 μg/mL) and 133 ± 8.6 μM (323 μg/mL) for C. krusei, 46 ± 2.2 μM (111 μg/mL) and 141 ± 7.1 μM (342 μg/mL) for C. parapsilosis, and 24 ± 1.9 μM (59 μg/mL) and 86 ± 3.6 μM (210 μg/mL) for C. tropicalis.

**Figure 1:**
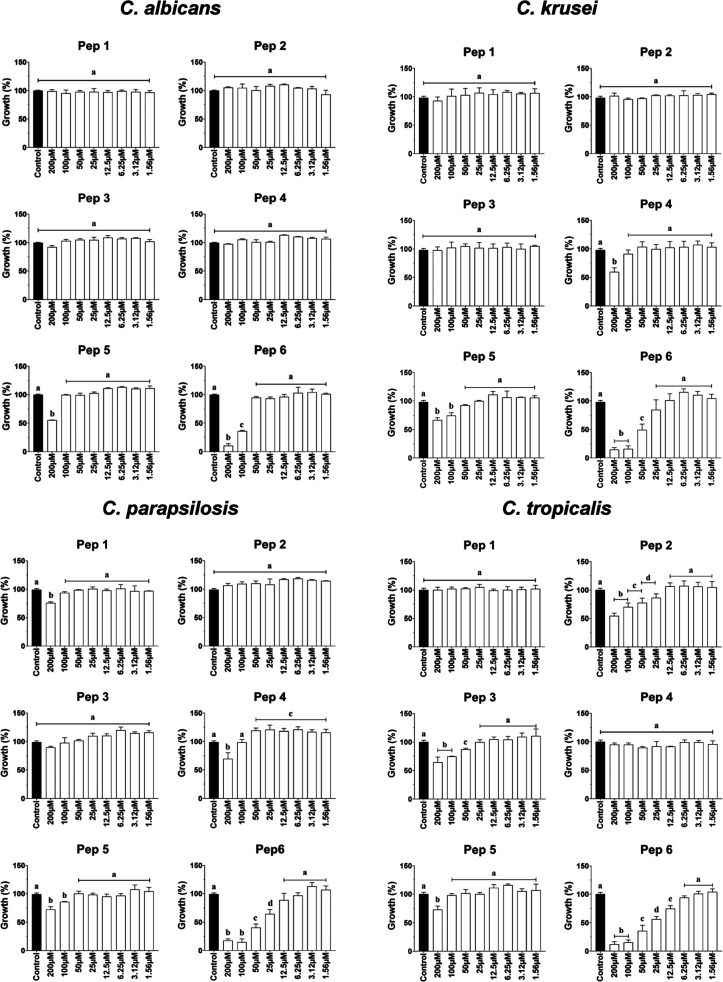
Anticandidal activity of floral nectar peptides. Pep 1 (VCPCACCSTPRRV), Pep 2 (EYCSAKSAKPGVHCRSCALVNMYK), Pep 3 (LHSGEGSTCYFFKTNPCCCGTWNCT), Pep 4 (WKCGSTPAPRKYCTCVAKMW), Pep 5 (HRFSYMCFVAQVLNKDYCSCCKF), and Pep 6 (KHYSCTRHGYCLACYKRWF), at varying concentrations, were evaluated for their inhibitory effects on C. albicans, C. krusei, C. parapsilosis, and C. tropicalis. The experiments were conducted three times, with each biological replicate executed in triplicate. All results were presented as the mean and corresponding standard deviation. Distinct letters denote significantly different groups (*p* < 0.05) from the control (0.15 M NaCl containing 5% DMSO).

The Antimicrobial Peptide Database (APD) presently comprises 5099 peptides (https://aps.unmc.edu/, accessed on 13 January 2025), which includes 3306 natural antimicrobial peptides (AMPs) sourced from the six kingdoms (410 from bacteria, 5 from archaea, 8 from protists, 29 from fungi, 268 from plants, and 2580 from animals), 1299 synthetic peptides, and 231 predicted AMPs with different functions or activities. APD contains 926 anticandidal peptides (https://aps.unmc.edu/, accessed on 13 January 2025). Antimicrobial peptides exhibit a wide range of biochemical properties and mechanisms of action, resulting in a significant diversity of biological activity. Consequently, comparing MIC and IC_50_ values, especially among different microorganisms, is exceedingly challenging. For example, ToAP2 and NDBP-5.7, which are two peptides discovered through the analysis of scorpion venom cDNA libraries, exhibited MIC values of 12 μM and 25 μM against C. albicans,^12^ respectively. In contrast, it was found that the leaf peptides of Capsicum annuum did not exhibit any inhibitory effect on C. parapsilosis and C. tropicalis.^13^

Perez-Rodriguez and colleagues^14^ examined the main biochemical characteristics and modes of action of 20 anticandidal peptides derived from diverse biological sources. The peptides ranged from 8 to 101 amino acids and exhibited MIC values from 0.18 to 800 μM.^14^ Noteworthy among the identified peptides is NaD1, which was isolated from the flowers of Nicotiana alata. It exhibits significant antifungal efficacy against many agronomically relevant filamentous fungi and both susceptible and resistant strains of C. albicans.^15,16^ NaD1 interacts with the fungal cell surface, leading to membrane permeabilization and the induction of excessive reactive oxygen species (ROS) production.^17,18^

Due to its higher inhibitory capacity, peptide Pep6 was selected for all subsequent assays involving characterization, mechanisms of action, and toxicity. Additionally, the peptide Pep6 was named nicotianin-I, referencing the first antimicrobial peptide identified in the floral nectar of a Nicotiana species. A novel experiment was conducted to assess the fungicidal or fungistatic properties of nicotianin-I. Cell viability tests, using MTT reagent, demonstrated that nicotianin-I exhibited fungicidal activity against C. albicans, C. krusei, C. parapsilosis, and C. tropicalis, with an efficacy of 83%, 79%, 81%, and 84%, respectively, at 200 μM (Figure S1). Other antimicrobial peptides possessing fungicidal properties against Candida strains have been documented, such as kyotorphin-derived conjugated peptides, which showed a minimum fungicidal concentration (MFC) of 1000 μM (IbKTP-NH2) against C. albicans and C. krusei,^19^ and temporin B-derived peptides, exhibiting fungicidal activity at 1.8 μM (IC_90_).^20^

Our findings also indicate that nicotianin-I diminished its efficacy at reduced doses (Figure S1). Interestingly, nicotianin-I-treated cells, at 12.5 μM, exhibited improved cell viability compared to control cells of C. albicans, C. parapsilosis, and C. tropicalis (Figure S1). MTT, a formazan salt, is reduced inside cells by oxidoreductases, dehydrogenases, or reduced coenzymes (NADH and NADPH).^21^ It is used to check whether cells are alive or how metabolically active they are. Under stress conditions, certain cells can augment the expression of stress-response proteins, demanding higher metabolic and energy investments.^22^ This may partially explain the augmented metabolism of Candida cells with reduced doses of nicotianin-I. Some studies examining cell viability in fungi,^23^ bacteria,^24^ and malignant (leukemia) cells^25^ also observed similar findings.

### Mechanism of Action

Among the six tobacco floral nectar peptides,^11^ nicotianin-I exhibits the lowest predicted DNA-binding activity (18.7%), a feature that could greatly impede its function as eDNA, a quorum-sensing molecule from the extracellular matrix of Candida cells, could imprison and inactivate it.^26^ On the other hand, Chen and Jiang^27^ described that the primary mechanism by which many antimicrobial peptides function is by interacting with the membrane of the target organism. Therefore, our hypothesis is that the anticandidal activity of nicotianin-I is linked to its interaction with negatively charged microbial membranes as it has a positive net charge (+6). This interaction could lead to disruption of the membrane, either by creating pores or through membrane micellization. Subsequent investigations were conducted to elucidate the mechanism of action by which nicotianin-I acts on fungal cells.

Candida cells exposed to nicotianin-I and propidium iodide (PI) exhibited red fluorescence (Figure 2a), while the control cells did not display any fluorescence. PI is a fluorophore that produces red fluorescence upon binding to DNA. Furthermore, it is impermeable to the healthy plasma membrane. Hence, the presence of red fluorescence indicates membrane damage,^28^ potentially involving the formation of pores. In addition, we conducted an experiment to investigate the formation of pores induced by nicotianin-I using fluorescein isothiocyanate conjugated with a 4 kDa dextran polymer (FITC-D4). In this case, the presence of green fluorescence within the cells indicates the formation of pores with diameters capable of crossing molecules with minimum molecular masses of 4 kDa. Only the species C. albicans and C. tropicalis, when treated with nicotianin-I, exhibited fluorescence raising. Compared with the control groups, the fluorescence increased by 45% for C. albicans and 76% for C. tropicalis. There was no increase in green fluorescence inside C. krusei and C. parapsilosis (Figure 2b).

**Figure 2:**
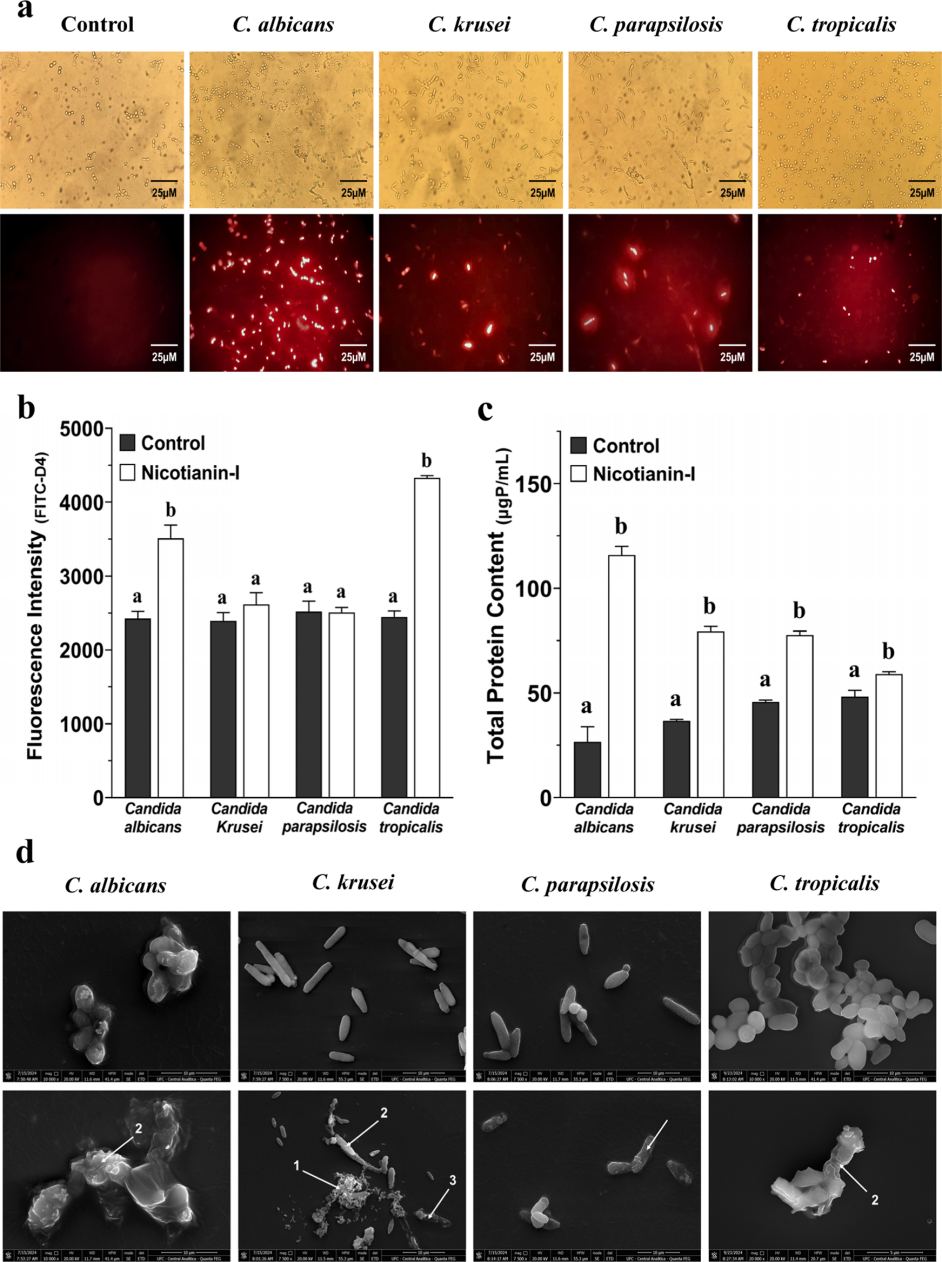
Analysis of membrane damage and pore formation. (a) Propidium iodide fluorescence observed after 24 h of incubation with nicotianin-I (excitation/emission: 488/525 nm). Superior panel: light microscopy. Bottom panel: fluorescence microscopy. Control: C. albicans cells subjected to 0.15 M NaCl containing 5% DMSO. (b) FITC-dextran (4 kDa) fluorescence was determined after 24 h of incubation with nicotianin-I. Fluorescence intensity was quantified using emission and excitation wavelengths of 490 and 520 nm, respectively. (c) Protein content of cell-free supernatant treated with nicotianin-I (IC_50_). (d) SEM images presenting (1) leakage of intracellular compounds, (2) rugous cells indicative of membrane damage, and (3) cellular debris, all caused by nicotianin-I after 24 h. Upper panel: control cells (0.15 M NaCl containing 5% DMSO). Bottom panel: Cells treated with peptide nicotianin-I (IC_50_). The experiments were conducted three times, with each biological replicate executed in triplicate. All results were presented as the mean and corresponding standard deviation. Distinct letters denote significantly different groups (*p* < 0.05) relative to the control (0.15 M NaCl containing 5% DMSO).

We also investigated the protein release by Candida cells following treatment with nicotianin-I to assess the membrane damage. Extracellular protein levels were significantly increased in all strains treated with nicotianin-I (Figure 2c). The extracellular protein levels increased by 335% for C. albicans, 117% for C. krusei, 70% for C. parapsilosis, and 22% for C. tropicalis. The protein outflow results for C. krusei and C. parapsilosis appear contradictory when contrasted with the experimental evidence of pore development (Figure 2b). However, the inability to maintain 4 kDa dextran probes within the cells indicates that nicotianin-I operates through detergent-like mechanisms rather than generating pores. This statement is corroborated by our ultrastructural morphological analyses conducted via scanning electron microscopy (SEM) (Figure 2d), which revealed that C. krusei and C. parapsilosis treated with nicotianin-I exhibited significant membrane damage, characterized by ruptures, intracellular content leakage, and cellular debris. Conversely, the SEM pictures of C. albicans and C. tropicalis exhibited increased rugosity relative to the control cells, potentially indicating pore development induced by the peptide treatment. Consequently, these findings indicate that nicotianin-I operates via a pore-forming mechanism in C. albicans and C. tropicalis and through a detergent-like lysis mechanism in C. krusei and C. parapsilosis. The diverse mechanisms of action across different species can be explained by the distinct phospholipid membrane compositions of each microorganism,^29^ which may result in different oligomerization patterns of nicotianin-I. Nonetheless, additional research is necessary for accurately determining the membrane-binding characteristics of nicotianin-I on various cell membrane types.

Coupled with membrane damage, the mechanism of action of AMPs also is commonly associated with ROS overproduction.^30^ ROS are subproducts of metabolism, and their accumulation is commonly associated with enhanced metabolic response of cells under stress conditions, signaling for metabolic adjustments and/or programmed cell death.^31,32^ Therefore, DCFH-DA assays were performed to evaluate the ROS overproduction induced by nicotianin-I. This fluorescence probe is deacetylated by cell metabolism (forming DCFH) and oxidizes (fluorescent DCF^–^) in the presence of most ROS, presenting optimal coverage for oxidative stress appraisal, indicating superoxide anions, hydrogen peroxide, peroxyl, and hydroxyl radicals.^31^

Candida cells treated with nicotianin-I exhibited green fluorescence (Figure 3a), indicating an ROS accumulation. Similarly, a quantitative evaluation correlated greater oxidative stress with higher concentrations of nicotianin-I (Figure 3b). Incrementations on fluorescence intensity accounted for nicotianin-I at its IC_50_ and 2-fold IC_50_: 108%/303% for C. albicans, 220%/280% for C. krusei, 88%/281% for C. parapsilosis, and 81/238% for C. tropicalis. ROS overproduction may cause damage to multiple cellular components and lead to cell death.^30^ In this case, the antifungal capacity of nicotianin-I might act by membrane damage and oxidative stress. These mechanisms of action have been related to other AMPs.^33^

**Figure 3:**
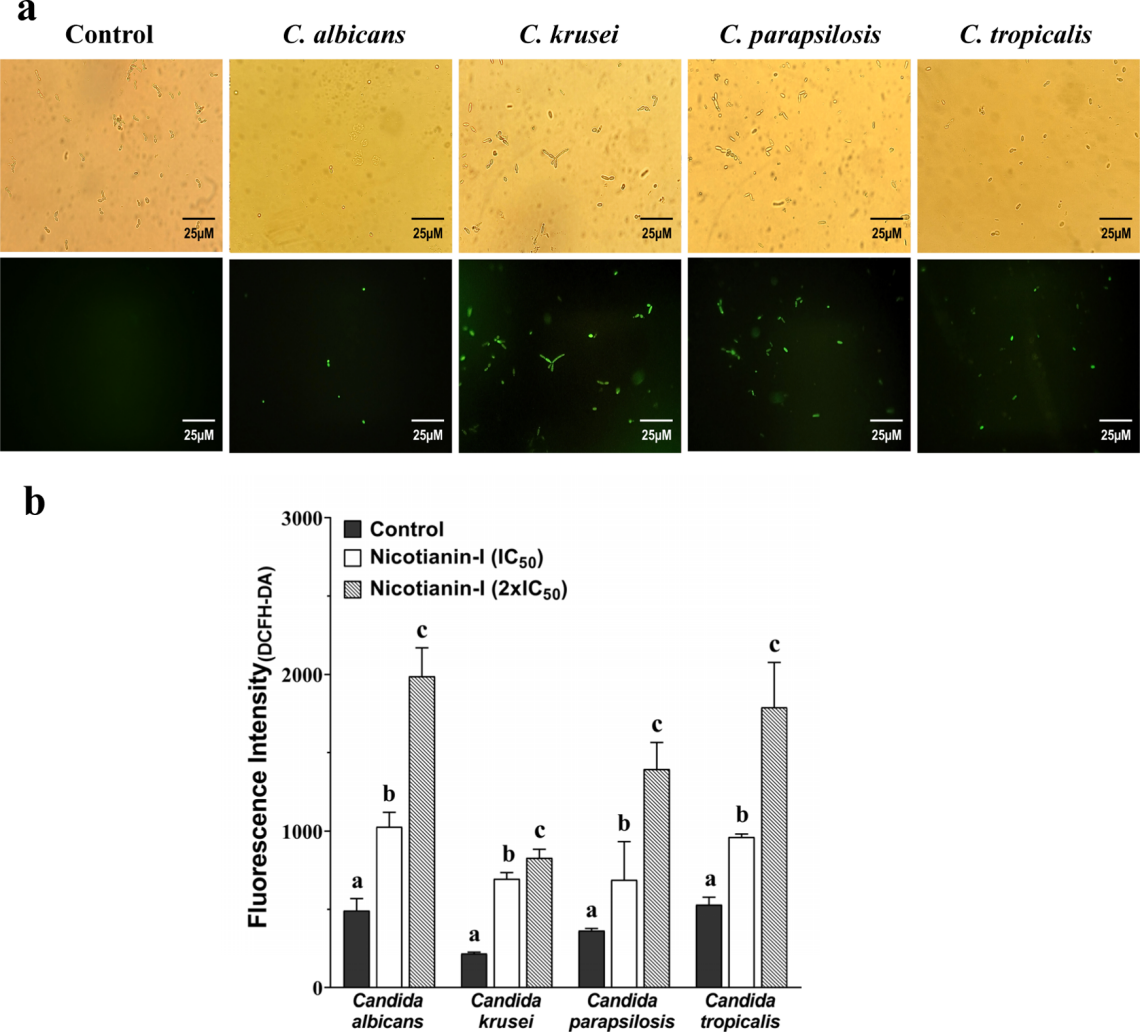
Assessing ROS overproduction induced by nicotianin-I. (a) DCFH-DA fluorescence of nicotianin-I (IC_50_)-treated cells (3 h) was observed by fluorescence microscopy (excitation/emission: 485/530 nm). Upper panel: light microscopy. Bottom panel: fluorescence microscopy. Control: C. parapsilosis cells treated with 0.15 M NaCl containing 5% DMSO. Green fluorescence indicates ROS overproduction caused by nicotianin-I. (b) DCFH-DA fluorescence quantification of yeast cells treated with nicotianin-I (IC_50_ and 2 × IC_50_) (excitation/emission: 485/530 nm). The experiments were conducted three times, with each biological replicate executed in triplicate. All results were presented as the mean and corresponding standard deviation. Distinct letters denote significantly different groups (*p* < 0.05) relative to the control (0.15 M NaCl containing 5% DMSO).

### Structural Characterization

The circular dichroism (CD) spectra of nicotianin-I demonstrate variability across different pH values (Figure 4a and Table 1). At pH 5.0 and 7.0, the CD spectra of nicotianin-I exhibit two typical characteristics of a polyproline II (PPII) helix: a pronounced negative band in the range of 190–200 nm and a weaker positive band at 220–230 nm.^34^ The structure is identified as a left-handed helix, exhibiting a dihedral angle usual of β-strands, and possesses an overall shape like a triangular prism.^35^ PPII was identified in the collagen triple helix. However, other circular dichroism studies indicate that PPII may be present in various folded proteins.^35^ Nicotianin I notably lacks proline residues, but other studies indicate that proline residues are not strictly necessary for the formation of PPII, thereby establishing PPII as a distinct category within secondary structures.^36^

**Figure 4:**
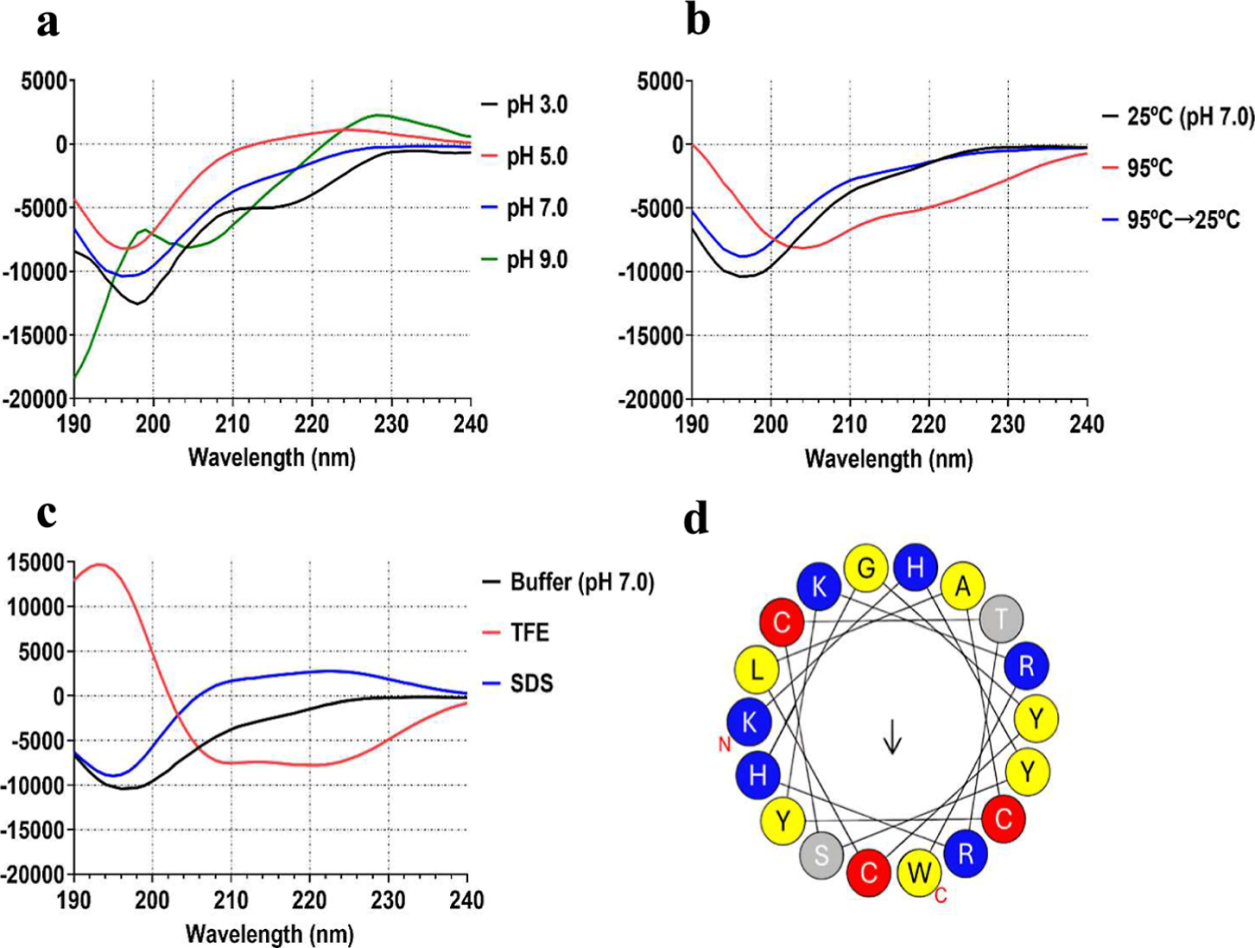
Secondary structure of nicotianin-I measured by circular dichroism (CD) under different conditions. (a) CD spectra of nicotianin-I at pH 3.0, 5.0, 7.0, and 9.0. (b) CD spectra of nicotianin-I at 25 °C, after heating up to 95 °C, and after heating up to 95 °C and cooling to 25 °C. (c) CD spectra of nicotianin-I in the presence of 20 μM SDS or 50% TFE. All analyses used the peptide at 50 μM. (d) Helical wheel projection was created using the HeliQuest program (https://heliquest.ipmc.cnrs.fr/cgi-bin/ComputParamsV2.py). The black arrow indicates the direction and intensity of the hydrophobic moment. Amino acid residues are depicted as follows: hydrophobic (yellow), positively charged (blue), uncharged polar (gray), and cysteine (red).

**Table 1:** Nicotianin-I Secondary Structure Distribution at Different pHs and Temperatures and in the Presence of SDS or TFE

solution	α-helix (%)	β-structures (%)	others
sodium phosphate buffer pH 3.0	–1.0	26.0	75.2
sodium acetate buffer pH 5.0	–2.6	30.5	72.1
sodium phosphate buffer pH 7.0	0.3	30.5	69.2
Tris-HCl buffer pH 9.0	3.3	31.4	65.3
25 °C	0.3	30.5	69.2
90 °C	8.9	36.2	54.9
90 °C → 25 °C	0.6	36.7	62.6
10 μM SDS	1.7	35.6	62.7
50% TFE	29.4	42.3	28.3

A significant spectral shift was observed between pH 7.0 and 9.0, indicating a pH-sensitive conformational transition. This behavior can be explained by the ionization of side chains with p*K*_a_ values in this range. The three cysteine residues in the peptide may experience deprotonation of their thiol groups (p*K*_a_ 8.2), which could affect the local conformation or facilitate disulfide bond formation under oxidizing conditions. Consequently, it remains uncertain whether one or all three cysteines are deprotonated as a result of the pH increase. The elucidation of nicotianin-I’s structure through NMR measurements would address this issue. Furthermore, other ionizable residues, including histidine (p*K*_a_ 6.0), lysine (p*K*_a_ 10.5), and arginine (p*K*_a_ 12.5), may contribute to these structural changes by modifying electrostatic interactions, solvation, and backbone flexibility. The pH-induced shifts demonstrate the structural plasticity of nicotianin-I and its ability to adopt different conformations in response to changes in the microenvironment.

The thermal stability of nicotianin-I and its renaturing capacity were evaluated and compared to the CD spectra of the control (peptide at pH 7.0 at 25 °C). Heating to 95 °C resulted in changes in the CD spectrum, characterized by a significant reduction in ellipticity, suggesting a conformational transition. After cooling to 25 °C, the CD spectrum was restored, demonstrating the reversibility of the conformational change (Figure 4b). A temperature ramp experiment was conducted to investigate this behavior in detail, revealing a gradual transition and a melting temperature (*T*_m_) of approximately 48.8 °C (data not shown). The findings indicate a reversible structural adjustment rather than classical denaturation, likely attributed to moderate conformational flexibility, a characteristic also noted in other peptides.^37^ The observed reversibility enhances the conformational resilience of nicotianin-I and may indicate a beneficial characteristic for its antimicrobial activity.

When subjected to a membrane-mimetic environment, simulated by TFE, the nicotianin-I spectrum established a typical α-helicoidal “W” pattern (Figure 4c), with minimum values of around 209 and 221 nm.^38^ Structural analysis revealed an increased percentage of the α-helix content (29.4%) relative to aqueous solutions (Table 1). This change can be due to TFE’s capacity to diminish peptide–water hydrogen bonding and enhance intramolecular hydrogen bonds, thus stabilizing helicoidal structures.^39^ Nonetheless, TFE-induced helicity may not accurately represent the peptide’s natural structure under physiological environments. Instead, it emphasizes the peptide’s structural capacity to assume an α-helical conformation in hydrophobic or membrane-mimicking conditions. Comparable behavior has been seen in many AMPs,^39,40^ which assume helical conformations following membrane insertion, consequently augmenting their membrane-permeabilizing ability.^39^ Conversely, exposure to sodium dodecyl sulfate (SDS), an environment mimicking negatively charged micelles, did not yield a notable increase in the α-helical content (Figure 4c). Therefore, the spectral profile in SDS indicates that nicotianin-I may retain a certain level of organized extended structure similar to polyproline type II helix.

Various antimicrobial peptides possess amphipathic α-helical structures, distinguished by different hydrophobic and hydrophilic regions, which are crucial for their activity.^41^ To define these areas, the helical wheel projection has been used to show where the hydrophobic and hydrophilic residues are along the α-helix.^42^ The helical wheel projection shows that nicotianin-I’s hydrophobic and hydrophilic residues are spread out along the α-helix (Figure 4d). It lacks a clearly defined hydrophobic face. This elucidates its low hydrophobic moment (0.215 μH) and supports a possible polyproline II helical structure as the helical wheel projection suggests the absence of an amphipathic driver for an α-helix conformation. This result is important as it will facilitate the development of novel analogues with better antimicrobial effectiveness. This could be done by adding positively charged amino acids, preferably to the hydrophilic side of the peptide, or by replacing polar amino acids on the hydrophobic side of the helix with hydrophobic ones. This method was utilized to synthesize five new peptides derived from the antimicrobial peptide temporin F.^43^ The peptides demonstrated improved activity against both Gram-negative and Gram-positive bacteria. Moreover, they demonstrated enhanced resistance to enzymatic degradation.^43^ Similarly, to improve the antimicrobial activity of Uperin 3.6, three analogues were created by substituting less hydrophobic amino acids with cationic lysine residues. The net positive charge of peptides was augmented, maintaining their amphipathic structures, which led to a decrease in MIC from 64–128 mg/L to 16–64 mg/L. Peptides exhibited minimal hemolytic action and considerable toxicity toward two normal human epithelial cell lines.^44^

### Cytotoxic Activity

#### In Vitro Assays

Erythrocytes and mammal cell lineages are commonly used to evaluate indicatives of cytotoxicity by a possible new pharmacological candidate.^45^ All of the cytotoxicity assays of nicotianin-I were conducted and compared to Pep1, which presented no anticandidal activity (Figure 1). The hemolytic assays using human erythrocytes showed that Pep1 and nicotianin-I, both at 200 μM, presented a maximum percentage of hemolysis of 11.7% and 28.1% (Figure 5a), respectively. At concentrations between the calculated IC_50_ values for the tested yeast, the hemolytic activity of nicotianin-I was about 6.3%. These results pair with or surpass others regarding antimicrobial peptide cytotoxicity. For example, Rana frog’s derived antimicrobial peptides have exhibited hemolysis values ranging from 20 to 100% at 100 μM.^46^

**Figure 5:**
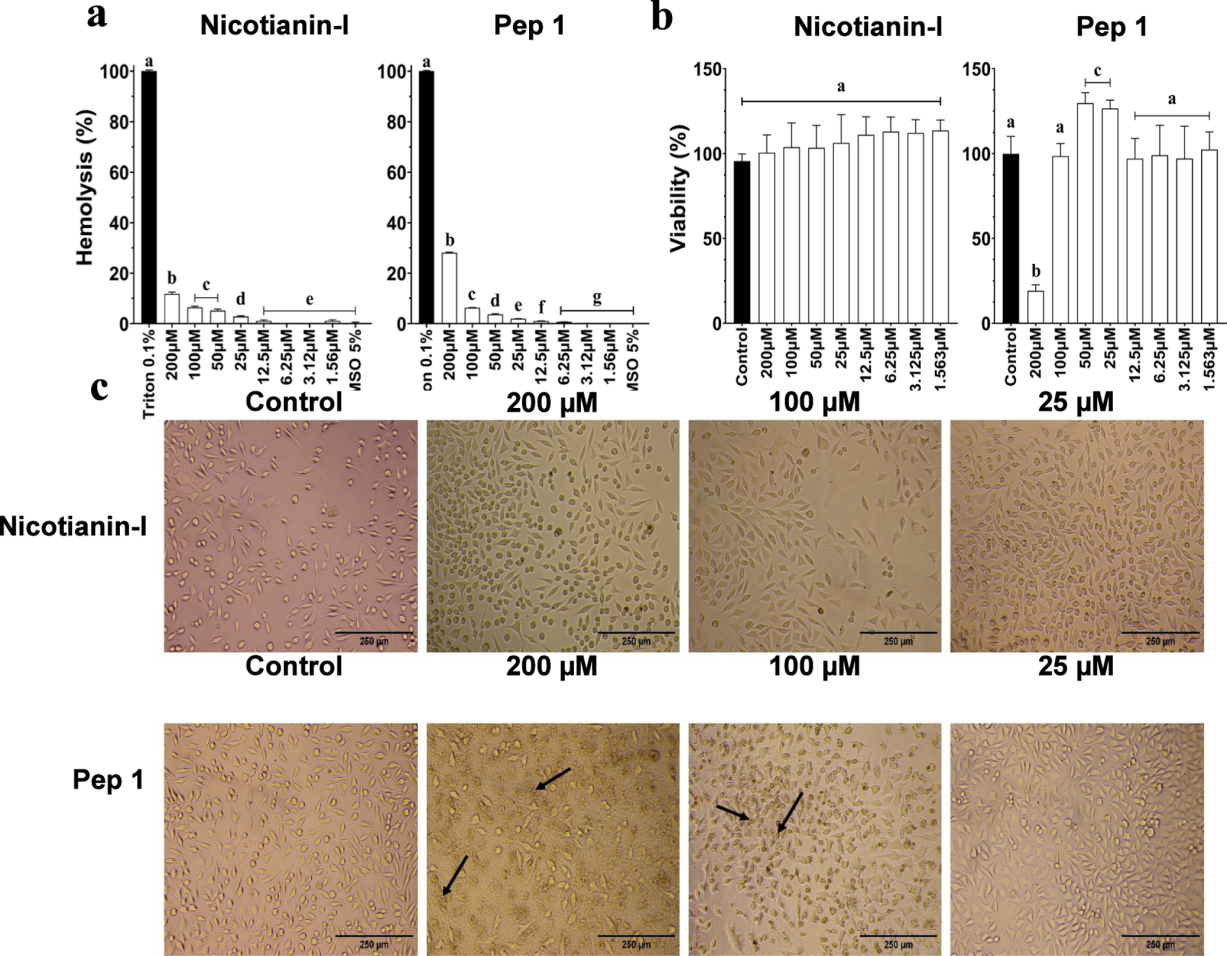
Cytotoxicity of nicotianin-I against human erythrocytes and murine fibroblast cells. (a) Hemolysis percentage of nicotianin-I and Pep1 regarding positive control (0.1% Triton-X-100). (b) Murine fibroblast cell viability (MTT) results. (c) L929 cells treated with different concentrations of nicotianin-I after 24 h of incubation. Images were obtained by a phase contrast optic microscope (200× magnification). Arrows point to abnormal cells and cell debris. Control: cells treated with 0.15 M NaCl containing 5% of DMSO. The experiments were conducted three times, with each biological replicate executed in triplicate. All results were presented as the mean and corresponding standard deviation. Distinct letters denote significantly different groups (*p* < 0.05) relative to the control (0.15 M NaCl containing 5% DMSO).

The antiproliferative assay using L929 cells revealed that peptide Pep1 was capable of reducing the cellular viability up to 80.95% at 200 μM (Figure 5b). The cell damage was visually observed by the increased turbidity of the media and presence of cell debris by phase contrast microscopy (Figure 5c). On the other hand, nicotianin-I showed no cytotoxicity to fibroblasts at 200 μM (Figure 5b,c). Other studies have shown disparities between antiproliferative tests using fibroblast and hemolytic assays, as shown by THL-2-1, a de novo designed peptide.^47^

#### In Vivo Assays

This is, to the best of our knowledge, the third study assessing the toxicity of antifungal peptides in zebrafish. The first evaluated parameter was the impact of nicotianin-I on the survival rate of the zebrafish embryos. The survival rate following exposure to nicotianin-I for 96 h was 90% at 50 μg/mL (21 μM), comparable to the negative control rate of 95%. At the maximum dose evaluated (100 μg/mL or 42 μM), zebrafish embryos exhibited a survival rate of 40%. The only lethal effect detected was embryo coagulation. Embryo coagulation rates of up to 25% may be regarded as typical, reflecting the spontaneous mortality of zebrafish embryos occurring 24 h postfertilization.^48,49^ The LC_50_ for nicotianin-I was determined to be 131 μg/mL or 54 μM. Substances exhibiting embryotoxicity at concentrations beyond 100 μg/mL in zebrafish are usually considered as having minimal toxicity since testing higher concentrations may surpass the osmotic threshold.^50^

For instance, three effective mastoparan analogue peptides targeting C. albicans demonstrated LC_50_ values in zebrafish embryos between 9.6 and 15.6 μg/mL.^51^ Likewise, PepM3, an IsCT peptide analogue obtained from the venom of Opisthacanthus madagascariensis exhibiting anticandidal properties, revealed an LC_50_ of 125 μg/mL in zebrafish embryos, which the authors consider indicative of minimal toxicity.^52^ Nicotianin-I exhibits low toxicity in this model as its LC_50_ (131 μg/mL) is equivalent to or exceeds that of other antifungal peptides.

The LC_50_ of nicotianin-I is clearly greater than those of commercial antifungal drugs. Amphotericin B (AmB) and nystatin (Nys) exhibited LC_50_ values of 1.3 and 6.2 μg/mL, respectively, in zebrafish embryos at 120 h postfertilization.^53^ However, the MICs for Nys (1 μg/mL) and AmB (0.25 μg/mL) against C. albicans and C. parapsilosis are significantly lower than those of nicotianin-I, which are 292 and 342 μg/mL, respectively. The therapeutic index (the ratio of toxicity to efficacy) of nicotianin-I is 0.45 for C. albicans and 0.38 for C. parapsilosis, whereas for AmB and Nys, the indices are 5.2 and 6.2, respectively.

Concerning the nonlethal effects, hatching delay (hd) and granules around the embryo (gae) were noted in the presence of nicotianin-I (Figure 6j–l) but exclusively at the highest dose examined (100 μg/mL). Thus, the lowest observed adverse effect level (LOAEL), defined as the minimal concentration or quantity of a drug identified by experimentation or observation to induce an undesirable impact, was 100 μg/mL (Table 2). Notwithstanding the occurrence of these effects, they were transient and hence did not influence embryo development further. On the other hand, Nys and AmB induced inner organ toxicity and various developmental abnormalities in a dose-dependent manner, leading to lethal outcomes. Zebrafish embryos exhibited cardiac abnormalities, including pericardial edema and reduced heartbeat, following exposure to 3 μg/mL of Nys (3 × MIC) and 0.8 μg/mL of AmB (3.2 × MIC). Furthermore, the administration of both drugs at subtherapeutic doses (<1 × MIC) resulted in a reduction of yolk absorption, suggesting a potential hepatotoxic effect.^53^ While both polyenes exhibit significantly higher therapeutic indexes compared to nicotianin-I, it is important to note that cytotoxicity remains a common and undesirable side effect of antifungal agents. In addition, zebrafish embryos exhibit greater sensitivity to toxicants compared with traditional mammalian models, potentially resulting in an overestimation of toxicity levels. In future studies, we will evaluate the toxicity of nicotianin-I in rodents to develop a comprehensive safety profile, facilitating a more precise calculation of its therapeutic index.

**Figure 6:**
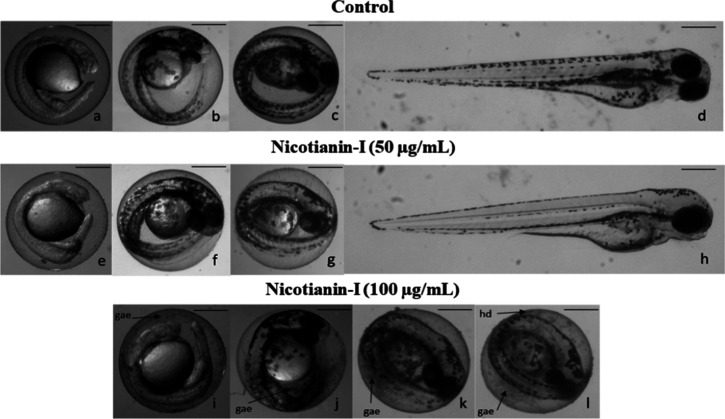
Effect of nicotianin-I on zebrafish embryos and larvae. (a–d) Zebrafish embryos and larvae with normal development after 24, 48, 72, and 96 hpf exposed only to E3 medium (control), respectively. (e–h) Zebrafish embryos and larvae with 24, 48, 72, and 96 hpf exposed to 50 μg/mL of nicotianin-I, respectively. (i–k) Zebrafish embryos with 24, 48, and 72 hpf, respectively, exposed to 100 μg/mL of nicotianin-I, showing granules around the embryos (gae). (l) Zebrafish embryo with 96 h exposure to 100 μg/mL of nicotianin-I, showing granules around the embryos (gae) and hatching delay (hd). Bars: 500 μm.

**Table 2:** Toxicological Effects of Nicotianin-I on Zebrafish Embryos^a^

embryo toxicological effects	nicotianin-I
eye malformation	n.e.^b^
otolith malformation	n.e.
mouth malformation	n.e.
spine malformation	n.e.
body pigmentation	n.e.
**hatching delay**	**100 μg/mL**
yolk sac edema	n.e.
pericardial edema	n.e
blood clotting	n.e.
undersize	n.e.
**granules around the embryo**	**100 μg/mL**
mortality (LC_50_)	131.85 μg/mL

a Bold values are LOAEL (lowest-observed-adverse-effect level).

b n.e.: no effect or less than 20% of embryos affected.

## Conclusions

Our findings were able to assert the antifungal efficacy of the ornamental tobacco’s AMPs, with a notable distinction for nicotianin-I, which exhibited antifungal potential against all Candida strains. Nicotianin-I promoted membrane permeabilization, possibly creating pores or inducing cell lysis, resulting in leakage of the cell contents. Additionally, it prompted the overproduction of ROS, indicating its diverse mechanisms of action. In terms of cytotoxicity to nontarget cells, it demonstrated a significantly low toxic profile to human erythrocytes, murine fibroblasts, and zebrafish embryos. Nicotianin-I exhibits several attributes that warrant additional exploration, regarding its potential as a therapeutic candidate. This AMP may also offer a promising amino acid framework for further bioinformatic enhancement, optimizing its advantages and mitigating its deficiencies.

## Materials and Methods

### Nectar Peptides

Nearly 800 peptides were found in the floral nectar of ornamental tobacco, a cross of N. sanderae and N. langsdorffii. Comprehensive bioinformatics and in vitro analyses identified six peptides with antimicrobial activity against phytopathogenic fungi and bacteria.^11^ Here, we evaluated the same six peptides against different Candida species of clinical interest. All peptides were synthesized by Synpeptide Co. Ltd. with purity above 95% using solid-phase peptide chemistry. The purity of the peptides was determined by reversed-phase, high-pressure liquid chromatography (RP-HPLC) and mass spectrometry. The peptides were Pep1 (VCPCACCSTPRRV), Pep2 (EYCSAKSAKPGVHCRSCALVNMYK), Pep3 (LHSGEGSTCYFFKTNPCCCGTWNCT), Pep4 (WKCGSTPAPRKYCTCVAKMW), Pep5 (HRFSYMCFVAQVLNKDYCSCCKF), and Pep6 (KHYSCTRHGYC LACYKRWF).

### Anticandidal Activity

The anticandidal activity was performed against the following species: C. albicans (ATCC 10231), C. krusei (ATCC 22019), C. parapsilosis (ATCC 6258), and C. tropicalis (INCQS 40042). The cell concentration was standardized by OD_600 nm_ to achieve a concentration of 1.5 × 10^3^ CFU/mL. The assays were performed using 96-well microplates, mixing the cell suspensions (100 μL) with 100 μL of each peptide serially diluted (200 to 1.56 μM, final concentrations) in 0.15 M NaCl containing 5% DMSO (dimethyl sulfoxide), which was used as a control. The plates were incubated for 24 h at 37 °C under agitation (150 rpm), and the readings were done at 600 nm using a Microtiter Plate Reader.^54^ The results are shown as the percentage of growth, considering the OD_600 nm_ of the control as 100%.

Cell viability assays also were performed using MTT (3-(4,5-dimethylthiazol-2-yl)-2,5-diphenyltetrazolium bromide) reagent.^13^ For that, cell suspensions (25 μL) were incubated with 25 μL of the most promising peptide (200–6.25 μM). After 24 h at 37 °C, 50 μL of 5 mg/mL MTT was transferred to each well and kept in the dark for 3 h. Afterward, 100 μL of 100% DMSO was transferred into each well and homogenized, and the OD_540 nm_ was measured using a microtiter plate reader.

### Membrane Damage Evaluation

The fluorophore propidium iodide (PI) was used to determine damage in the fungal cell membrane. First, nicotianin-I was incubated with cell suspensions at 37 °C for 24 h, as described before. After that, the samples were centrifuged at 5000*g* at 4 °C for 5 min, and the pellets (cells) were resuspended with 50 μL of 0.15 M NaCl containing 10 μL of 0.1 mg/mL PI. After 30 min at 25 °C in the dark, the samples were again centrifuged, and then the pellets were rewashed and rinsed in 50 μL of 0.15 M NaCl. The fluorescence was observed using a fluorescence microscope (Olympus System BX 41, Tokyo, Japan—an excitation wavelength of 488 nm and an emission wavelength of 525 nm).^55^

Further analyses were performed to determine the possible pore formation in the fungal cell membrane. For that, the same assays above were performed, adding fluorescein isothiocyanate (FITC) linked to 4 kDa dextran polymer instead of PI. The reading was done with the aid of a fluorescence microplate reader (Synergy MX, BioTek, Winooski, VT, USA) emission/excitation wavelengths of 490/520 nm, as described before.^55^ The pore formation also was evaluated by quantifying the extracellular protein content of the fungal cells treated with nicotianin-I. The cell-free supernatant (CFS) was collected after centrifugation (5000*g* at 4 °C for 5 min), and the soluble proteins were determined using the Bradford method^56^ for both the treated and nontreated cells.^57^

### Ultrastructural Analysis by Scanning Electron Microscopy (SEM)

The yeasts were incubated with nicotianin-I as described before. After that, the cells were washed three times with 0.15 M potassium phosphate buffer (pH 7.2) containing 2.5% glutaraldehyde. Finally, the material was dehydrated with ethanol serial concentrations (10–100%) before adding hexamethyldisilazane (HMDS) solutions (1:1 ratio and pure) and then transferred to sterilized circular coverslips (∼13 mm).^57^ The cells were coated on gold (QT150 ES, Quorum), and the captures were done (WD = 10 μm/mag = 10.000×/20.000×) using a scanning electron microscope (Quanta 450-FEG, FEI).

### Reactive Oxygen Species (ROS) Overproduction

To measure the ROS accumulation inside the cells treated with nicotianin-I, 2′,7′-dichlorofluorescein diacetate (DCFH-DA) was used as the fluorescence probe. The peptide was incubated with cell suspensions (1.5 × 10^6^) at 37 °C for 3 h using IC_50_ or 2-fold IC_50_. After that, the samples were centrifuged at 5000*g* at 4 °C for 5 min, and the pellets were resuspended with 50 μL of 0.15 M NaCl containing 9 μL of 0.1 mg/mL DCFH-DA. After 20 min at 25 °C in the dark, the samples were again centrifuged, and then the pellets were rewashed and rinsed in 50 μL of 0.15 M NaCl. The fluorescence was evaluated qualitatively by fluorescence microscope (Olympus System BX 41, Tokyo, Japan—an excitation wavelength of 485 nm and an emission wavelength of 530 nm) and quantitatively using a fluorescence microplate reader (Synergy MX, BioTek, Winooski, VT, USA) under the same excitation and emission wavelengths.^30^

### Structural Characterization

Circular dichroism (CD) spectra of nicotianin-I (50 μM) were achieved using a Jasco-815 spectropolarimeter (Jasco Inc., Tokyo, Japan) in a quartz cuvette (0.1 cm), ranging from 190 to 240 nm at 25 °C with 4 replicate scans per analysis. The structural stability was investigated under different pH values (3.0, 5.0, 7.0, and 9.0). The effect of temperature was evaluated by applying a temperature ramp (25–95 °C), wherein the temperature was incrementally raised by 3 °C per minute, with ellipticity monitored at a wavelength of 210 nm. Upon reaching 95 °C, the temperature was reduced back to 25 °C, and a new scan was performed to assess the potential renaturation of the peptide. Additionally, structural stability also was assessed in the presence of 20 μM sodium dodecyl sulfate (SDS) and 50% trifluoroethanol (TFE).^58^ Helical wheel projection was created using the HeliQuest program (https://heliquest.ipmc.cnrs.fr/cgi-bin/ComputParamsV2.py).^42^

### In Vitro Toxicity Assays

#### Hemolysis Assay

To assess a possible indicator of cytotoxicity, 150 μL of nicotianin-I, serially diluted with 0.15 M NaCl containing 5% DMSO, was incubated at 37 °C for 1 h with the same volume of 1% human erythrocyte (type O), which was obtained from the Hematology and Hemotherapy Center of Ceará, Brazil. Samples were centrifuged for 5 min at 2500*g*, and the supernatant was transferred to 96-well microplates. Released hemoglobin content was measured at OD_414 nm_. 0.1% Triton-X-100 and 0.15 M NaCl containing 5% DMSO were used as the positive and negative controls, respectively.^59^

#### Cytotoxicity Assay

L929 (murine fibroblast) cell lineage, kindly conceded by the Biological Physics Laboratory (Physics Department, Federal University of Ceará), was cultured on Dulbecco’s modified Eagle’s media (DMEM, Gibco) supplemented with 10% fetal bovine serum (FBS, Gibco), 10,000 U/mL penicillin, and 10 mg/mL streptomycin (Sigma-Aldrich Co., St. Louis, MO, USA) at 37 °C and 5% CO_2_ atmosphere saturation.^60^

Cell viability of peptide-treated fibroblast cells was tested by the MTT method. Shortly, L929 cells were trypsinized (trypsin–EDTA solution) and seeded into 96-well plates with DMEM (100 μL) at 1 × 10^5^ cells/mL for 48 h, followed by the addition of the same volume of serially diluted nicotianin-I (200–1.56 μM) and negative control (0.15 M NaCl). The images were captured with the aid of an optical microscope coupled with phase contrast (AE200, Motic, Barcelona, CT, ES) under 200× magnification. Finally, 10 μL of 5 mg/mL MTT was added to each well, and the mixture was incubated at 37 °C for 3 h. Formazan was solubilized with DMSO (150 μL), and the OD_570 nm_ was measured in a microplate reader.^61^

### In Vivo Toxicity Assays

#### Zebrafish Embryos

Zebrafish embryos (AB wild-type strain) with approximately 1 h postfertilization (hpf) were provided by the Production Unit for Alternative Model Organisms (UniPOM), Federal University of Paraiba (UFPB), João Pessoa, Brazil. A day before the experiment, zebrafish adults (male/female ratio of 2:1) were transferred to a 7 L spawning tank with a bottom mesh and a quick-opening valve for embryo collection. Embryos were collected on the day of the experiment and cultured in the adapted embryonic medium E3 (5.0 mM NaCl, 0.17 mM KCl, 0.33 mM CaCl, and 0.33 mM MgSO_4_) containing 0.005% methylene blue. Only spawnings with a fertilization rate ≥90% were used. Viable embryos (normal cleavage pattern and without morphological changes) were selected using an inverted light microscope (Televal 31, Zeiss, Germany) at 50× magnification. The experiments were approved by the Ethics Committee on the Use of Animals at UFPB, with authorization documented by protocol no. 4460140920.

#### Acute Toxicity Test

The Fish Embryo Acute Toxicity (FET test) test was conducted according to OECD’s guideline number 236 ^50^ adapted for 96-well plate, as described by Muniz et al.^62^ Zebrafish embryos with up to 3 hpf were exposed to five concentrations (6.25, 12.5, 25, 50, and 100 μg/mL) of nicotianin-I, with the last concentration being adopted as a limit concentration. Twenty wells were filled with 0.3 mL of E3 medium and 1 embryo. In the same conditions, E3 medium and 0.15 M NaCl containing 5% DMSO were used as controls.

Lethal and nonlethal effects were observed for 96 h. Embryos showing lethality end points (embryo coagulation, lack of somite formation, nondetachment of the tail bud, and lack of heartbeat) were considered dead. This number was used to determine the survival rate % [number of alive organisms/total number of organisms × 100] per tested concentration. The exposures were under static conditions (without renovation of the exposure solution). Observations were performed using a stereomicroscope at a 50× magnification. After 96 h, the surviving larvae were euthanized with eugenol and properly discarded.

### Statistical Analysis

All experiments were conducted three times, with each biological replicate executed in triplicate. All results were presented as the mean and corresponding standard deviation (SD). The ANOVA test was employed for multiple mean comparisons. Dunnett’s test was conducted to compare the means individually. All statistical analyses used a *p* < 0.05. GraphPad Prism 8.0 was used for statistical analyses and graphical production.

## Supplementary Material



## Notes

Effect of nicotianin-I on the cell viability of C. albicans, C. krusei, C. parapsilosis, and C. tropicalis (PDF)
