# Supplementary material for: Nicotianin-I: A Tobacco Floral Nectar Peptide with Anticandidal Activity
Source: ACS Omega. 2025 May 14;10(20):20213–25. doi: 10.1021/acsomega.4c10806 (PMC12120630; doi:10.1021/acsomega.4c10806)
Supplement: Supplementary file 1 [file ao4c10806_si_001.pdf]

Nicotianin-I: a tobacco floral nectar peptide with anticandidal activity

João M. M. Neto[1]; Tawanny K. B. Aguiar[1]; Mariana F. Oliveira[1]; Queilane L. S. G. Chaves[1]; Dário R. A. L. Mourão[1]; Viviane O. Silva[2]; Maria T. V. Nascimento[2]; Rômulo F. Carneiro[3]; Rafael X. Martins[4], Davi F. Farias[4], Brandon F. Sousa[5], Jeanlex S. Sousa[5]; Márcio V. Ramos[1], Cleverson D.T. Freitas[1*]

[1] Department of Biochemistry and Molecular Biology, Federal University of Ceará, Pici Campus, Fortaleza-Ceará, Brazil. CEP 60440-554.

[2] Department of Biology, Federal University of Ceará, Pici Campus, Fortaleza-Ceará, Brazil. CEP 60440-554.

[3] Department of Fishing Engineering, Federal University of Ceará, Pici Campus, Fortaleza-Ceará, Brazil. CEP 60440-554.

[4] Laboratory for Risk Assessment of Novel Technologies (LabRisk), Department of Molecular Biology, Federal University of Paraiba, Campus I, João Pessoa, Brazil. CEP: 58051-900

[5] Biological Physics Laboratory, Physics Department, Federal University of Ceará, Pici Campus, Fortaleza-Ceará, Brazil. CEP 60440-554.

*Corresponding author: Cleverson D.T. Freitas (cleversondiniz@ufc.br). Department of Biochemistry and Molecular Biology, Federal University of Ceará, Pici Campus, Building 907. Fortaleza-Ceará, Brazil. CEP 60440-554.

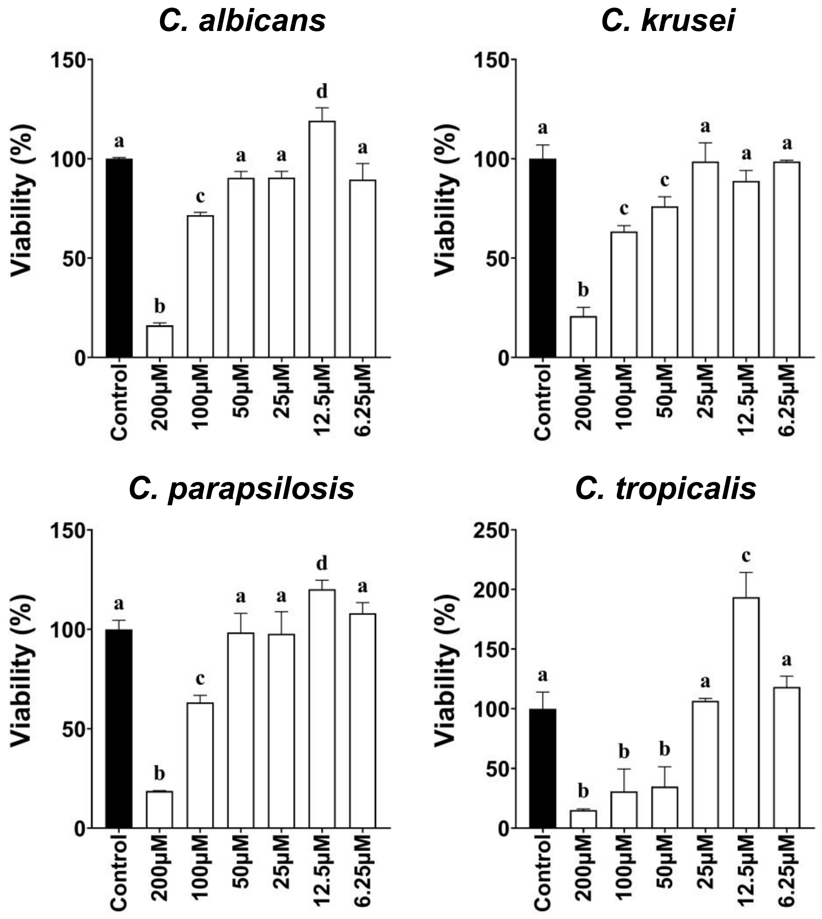

Figure S1. Effect of Nicotianin-I on the cell viability of *C. albicans, C. krusei, C. parapsilosis,* and *C. tropicalis*. The experiments were conducted three times, with each biological replicate executed in triplicate. All results were presented as the mean and corresponding standard deviation. Distinct letters denote significantly different groups ($p < 0.05$) relative to the control (0.15 M NaCl containing 5% DMSO).
